# **LncRNA LYPLAL1**,** miR-204-5p**,** and SIRT1: novel signatures for risk assessment of diabetic macrovascular complications**

**DOI:** 10.1038/s41598-024-75543-6

**Published:** 2024-10-15

**Authors:** Maysa A. Mobasher, Marwa A. Shabana, Mousa O. Germoush, Najlaa Yousef Abuzinadah, Amir Abd-elhameed, Shereen A. Baioumy, Moataz A. ElKot, Marwa M. Esawy

**Affiliations:** 1 Department of Pathology, Biochemistry Division, College of Medicine, Jouf University, 72388 Sakaka, Saudi Arabia; 2 Clinical Pathology Department, Faculty of Human Medicine, Zagazig University, Zagazig, Egypt; 3 Biology Department, College of Science, Jouf University, Sakakah, Saudi Arabia; 4 Department of biological science, College of Science, University of Jeddah, 23714 Jeddah, Saudi Arabia; 5 Internal Medicine Department, Faculty of Human Medicine, Zagazig University, Zagazig, Egypt; 6 Microbiology and Immunology Department, Faculty of Human Medicine, Zagazig University, Zagazig, Egypt; 7 Cardiology Department, Faculty of Human Medicine, Zagazig University, Zagazig, Egypt

**Keywords:** LncRNA LYPLAL1, MiR-204-5p, SIRT1, Diabetes, Macrovascular complications, Biochemistry, Biomarkers

## Abstract

Long-term, uncontrolled diabetes mellitus can lead to micro- and macrovascular problems. The protective function of lncRNA LYPLAL1 is to reduce endothelium cell inflammation by upregulating sirtuin 1 (SIRT1) and reducing microRNA (miR)-204-5p. This work attempted to examine the lncRNA LYPLAL1/miR-204-5p/SIRT1 molecules as diagnostic biomarkers for diabetic MVC and to assess their clinical correlations. The study enrolled 32 controls, 32 patients with diabetes alone, and 32 patients with diabetic MVC. RT-qPCR, or quantitative real-time PCR, was utilized to determine the expression levels of lncRNA and miR. SIRT1 was measured by ELISA. When comparing cases with MVC to those without MVC, the lncRNA LYPLAL1 and SIRT1 values were significantly lower. Conversely, patients with MVC had significantly higher miR-204-5p levels than those without MVC. The LncRNA LYPLAL1 performed best in terms of detecting MVC. It attained 90.6% specificity and 96.9% sensitivity. A combination of three markers (lncRNA LYPLAL1, miR-204-5p, and SIRT1) yielded the best accuracy at 98.4%. LYPLAL1 expression appeared to be an independent MVC predictor. Adjusted OR for LYPLAL1 expression was 405 (95% CI: 1.4–1200) (*p* = 0.039). When we compared cases with MVC to those without MVC, the lncRNA LYPLAL1 and SIRT1 values were significantly lower. Patients with MVC had significantly higher miR-204-5p levels than those without MVC. LYPLAL1 LncRNA demonstrated the best performance characteristics. LncRNA LYPLAL1 expression is an independent predictor of MVC.

## Introduction

Globally, the prevalence of diabetes has risen dramatically. Long-term, uncontrolled diabetes mellitus can lead to micro- and macrovascular problems that increase mortality and morbidity in those with the disease^1^. In Egypt, an estimated 10.9 million people have diabetes, which is anticipated to increase to 13 million by 2030 and 20 million by 2045. Egypt currently ranks tenth among countries with the greatest prevalence of diabetes and is anticipated to move up to ninth by 2045^2^. Diabetes-related vascular complications can affect multiple organs, such as the legs, brain, and heart (referred to as diabetes macrovascular complications, or MVCs), and microvascular problems associated with diabetes, which can affect the eyes, kidneys, foot, and nerves^3^. Heart disease and stroke account for 50% of the deaths of individuals with diabetes^4^. Reactive oxygen species are elevated in diabetes and prediabetes due to hyperglycemia and insulin resistance, which initiate intracellular molecular signaling. The development of macrovascular problems and atherosclerotic alterations is accelerated by the prothrombotic condition and the rise in inflammatory mediators^5^. Hence, it would be immensely helpful to have a basic understanding of the molecular mechanisms that lead to endothelial injury in hyperglycemia and inflammatory scenarios to rationally develop a medication for diabetic MVC^6^.

A family of non-coding single-stranded RNAs that are over 200 bases in length is known as long non-coding RNAs (lncRNAs), and they are thought to have no apparent role in coding proteins^7^. Over 200 diseases have been linked to dysregulated or malfunctioning lncRNAs, according to mounting data. The literature on diabetes is still accumulating new relationships^8^.

Research on the relationship between lncRNAs and the emergence of problems related to diabetes started^9^. The identification of numerous potential lncRNA loci is made possible by advancements in sequencing and microarray technologies, which also hasten the advancement of this field of study^10,11^. In recent years, increasing evidence has shown a growing list of lncRNAs associated with glucose homeostasis and diabetic consequences. The molecular mechanisms of lncRNAs can regulate target genes by acting as miRNA inhibitors, hence influencing the expression of protein-coding genes^12^. Despite the fact that several research has investigated the association between lncRNA and vascular diseases^13,14^, it is yet unclear what part of lncRNAs is implicated in the development of vascular anomalies in diabetes^15,16^.

Overexpression of lncRNA LYPLAL1-DT may reduce monophage adherence to endothelial cells (EC) surfaces, hence increasing the release of anti-inflammatory cytokines IL-10 and IL-13, providing additional evidence of EC protective properties. LncRNA LYPLAL1-DT alleviates the influence of hyperglycemia and inflammatory conditions on the proliferation, migration, autophagy, apoptosis, and inflammatory response of ECs^17^. These findings imply that lncRNAs play a role in regulating endothelial cells during diabetes-induced vascular injury and are a key component in the regulation of diabetic macrovascular problems^18^.

Zhu et al.^19^ reported that lncRNA LYPLAL1 has protective properties and is transported by exosomes from leukocytes to EC. The protective benefits of lncRNA LYPLAL1 via suppressing microRNA (miR)-204-5p and upregulating sirtuin 1 (SIRT1) reduce inflammation and encourage autophagy in EC. SIRT1 has also been shown to protect ECs from high glucose-induced damage by decreasing monocyte adherence to the vascular endothelium and is involved in the inhibitory effects of endothelial cell death^20^. SIRT1 may also exert antidiabetic effects via the modulation of insulin secretion and improvement of insulin resistance via its regulatory effects on insulin signaling, inflammation, mitochondrial function, and circadian rhythms^21^. These findings imply that these markers might be brand-new, prospective diagnostic markers as well as goals for the medical treatment of diabetes MVC^19^.

More than 50% of diabetic patients die of diabetic MVC such as cardiovascular disease, making it a major cause of morbidity and mortality. Early prediction of diabetic MVC will be helpful. Measuring lncRNAs expression levels can help with disease diagnosis, prognosis, and therapy planning. The protective effects of lncRNA LYPLAL1-DT against diabetic MVC by inhibiting miR-204-5p and upregulating SIRT1 were evaluated. This suggests that these markers might be effective risk predictors of diabetic MVC. So, this work attempted to examine the lncRNA LYPLAL1, miR-204-5p, and SIRT1 molecules as diagnostic biomarkers for diabetic MVC and to assess their clinical correlations.

## Subjects and methods

### Study design and subjects

Between October 2023 and January 2024, Zagazig University Hospitals served as the site of this case-control study. The Institutional Review Board (IRB) of the Faculty of Human Medicine at Zagazig University (IRB number #11155), which was established in accordance with the Declaration of Helsinki, verified the study. Every patient was randomly selected from the internal medicine and cardiology outpatient clinics. To take part in the study, each participant signed an informed consent form. Patients with cancer, chronic renal disorders, inflammatory diseases, and CVD diagnosed prior to diabetes mellitus were not included in the study. Prior to the study, the Epi Info software package 6 (Atlanta, Georgia, USA) was used to estimate the sample size. Given that the miR-204-5p expression levels in cases were 0.7 ± 0.45 and in controls were 1 ± 0.4, the sample size that was calculated with 80% power and a 95% confidence interval (CI) is 96 participants. There were 32 patients with diabetes alone, 32 patients with diabetic MVC, and 32 controls.

Patients were matched with healthy people based on their age, gender, and body mass index (BMI). The case of type 2 diabetes was identified using the standards established by the Americans based on A1C or plasma glucose criteria^22^. Macrovascular complications (MCV) included peripheral vascular disorders (PVD), cerebrovascular disease (CVD), and coronary artery disease (CAD). Patients with MVC included 20 patients with CAD, 4 patients with PVD, and 8 patients with CVD.

Several non-invasive tests were used to diagnose CAD, such as computed tomography angiography (CTA), single-photon emission computed tomography (SPECT), and positron emission tomography (PET). Nuclear stress testing, myocardial scintigraphy, and stress echocardiography were performed as functional non-invasive methods. When non-invasive testing is unsatisfactory or suggests a high chance of an incident, invasive testing using coronary angiography (ICA) was done^23^. To diagnose PVD, the ankle-brachial index (ABI), duplex ultrasonography (DUS), digital subtraction catheter angiography (DSA), CTA, and magnetic resonance angiography (MRA) were utilized^24^. Cerebrovascular disease (CVD) was diagnosed using many techniques such as Cerebral Angiography or imaging techniques such as carotid duplex, carotid ultrasonography, and magnetic resonance imaging (MRI)^25^.

### Methods

#### Sample preparation

Every participant provided their complete medical history and underwent a comprehensive clinical assessment. Each was informed about the 12-hour fast. After an 8-hour fast, one EDTA tube and two plain tubes were used for the collection of whole blood (BD Vacutainer^®^, Becton Dickinson & Co., NJ). The HbA1c test was conducted using the EDTA tube. A plain tube was used to separate serum for measuring insulin, glucose, and serum SIRT1 levels, and the serum that separated from the second plain tube was used to investigate the expression of miR and lncRNA. After fasting for a further four hours, a second sample was placed in a plain tube for the purpose of analyzing the lipid profile study. After collecting the plain tubes and leaving them to coagulate for half an hour at room temperature, the serum was separated using a 10-minute 1200 x g centrifugation.

#### Routine laboratory tests

The Cobas 8000-C702 Modular Analyzer (Roche, Germany) was used to do routine biochemical tests such as glucose as well as total cholesterol, high-density lipoprotein cholesterol (HDL-C), low-density lipoprotein cholesterol (LDL-C), and triglycerides that are components of lipid profiles. The Cobas 6000-C501 Modular Analyzer, manufactured by Roche in Germany, was used to calculate the glycated hemoglobin (HbA1C) percentage. The insulin levels were measured using an enzyme-linked immunosorbent assay (ELISA) kit from SunRed Biotechnology in Shanghai, China (Catalogue #: 201-12-0011). A formula involving the use of fasting insulin and fasting glucose was used to estimate the homeostasis model assessment parameter of insulin resistance (HOMA-IR)^26^.

#### SIRT1 measurement

The RayBio^®^Human SIRT1 ELISA Kit (RayBiotech, Norcross, Georgia, USA) (Catalogue #: ELH-SIRT1) was utilized to assess the serum SIRT1 levels. The absorbance was measured at 450 nm. A Sunrise absorbance reader was used to estimate the optical density (Tecan Trading AG, Männedorf, Switzerland). A standard curve is prepared from seven standards. The 1.2–300 ng/mL test standard range is used to detect SIRT1.

#### RNA extraction

All RNA from serum samples was extracted using the miRNeasy Serum/Plasma Kit, according to the instructions given by QIAGEN (GmbH, Hilden, Germany). The quantity and purity of the RNA were assessed using a NanoDrop-2000 spectrophotometer from Thermo Scientific (USA).

#### cDNA synthesis

One microgram of RNA was used in each reverse transcription reaction, which was carried out following the steps outlined by QIAGEN for the miScript RT II kit (GmbH, Hilden, Germany). The miScript SYBR Green PCR kit was utilized on a StepOne real-time PCR instrument that was manufactured by Applied Biosystems in the United States.

#### Quantitative real-time PCR

To find out how much lncRNA and miR were expressed, we used quantitative real-time PCR (RT-qPCR). The reaction mixture had a volume of 20 µL. The qRT-PCR reactions were runed in duplicate to provide reliable and accurate measurements of gene expression levels, reduce error, increase confidence in the results, and improve sensitivity. The RT-qPCR thermal included a 10-minute initial denaturation process at 95 °C, 15 s at 95 °C, 30 s at 58 °C, and 30 s at 70 °C, with a total of 40 amplification cycles commencing from the second step. An examination of the annealing curve confirmed the reaction’s specificity.

To normalize the expressions, GAPDH and U6 were used as reference genes. The relative expression of the lncRNA and miR was estimated using the 2-ΔΔCT calculation method. Primer sequences used in the real-time PCR were as follows: LYPLAL1-AS1 forward primer: 5’- GAGGAGGAGAAGCAAACTACAG-3’; LYPLAL1-AS1 reverse primer: 5’- GACTCAGTCATGCCACTAAGG-3’; miR-204-5p forward primer: 5’- CGCGTTCCCTTTGTCATCCT − 3; miR-204-5p reverse primer: 5’- AGTGCAGGGTCCGAGGTATT − 3; GAPDH forward primer: 5’-AC CAGGAAATGAGCTTGACA-3; GAPDH reverse primer: 5’-GACCACAGTCCATGCCATC-3; and U6 forward primer: 5’- ACACGCACAAACGAGAAAGG-3; U6 reverse primer: 5’- AGTGCAGGGTCCGAGGTATT-3.

### Statistical analysis

The data in this study do not follow a normal distribution. Multiple variables were compared using the Kruskal-Wallis H test and the Dunn’s test, a post hoc test. When necessary, we utilized the Chi-squared test and the Mann-Whitney U test. The cutoff value was determined using a Receiver Operating Characteristic (ROC) study. The association between variables was established using Spearman’s correlation coefficient technique. To quantify the risk, the odds ratio was calculated using logistic regression analysis. SPSS 20.0 (Chicago, IL, USA) was the statistical program used in this study, and in instances where the test resulted in statistical significance, the p-value was < 0.05.

## Results

### Characterization of subjects

Coronary artery disease accounted for the majority of MVCs, at 62.5%. In diabetic cases with and without MVC, the lipid profile and glucose profile were considerably higher than controls (P-value < 0.001), except for HDL-C values, which were lower (P-value = 0.007 and P-value < 0.001). HbA1c was considerably higher in MVC cases than in non-MVC cases (P-value = 0.016). Patients with MVC had significantly lower HDL-C values than those without MVC (P-value = 0.03) (Table 1).

### Markers levels

**Fig. 1:**
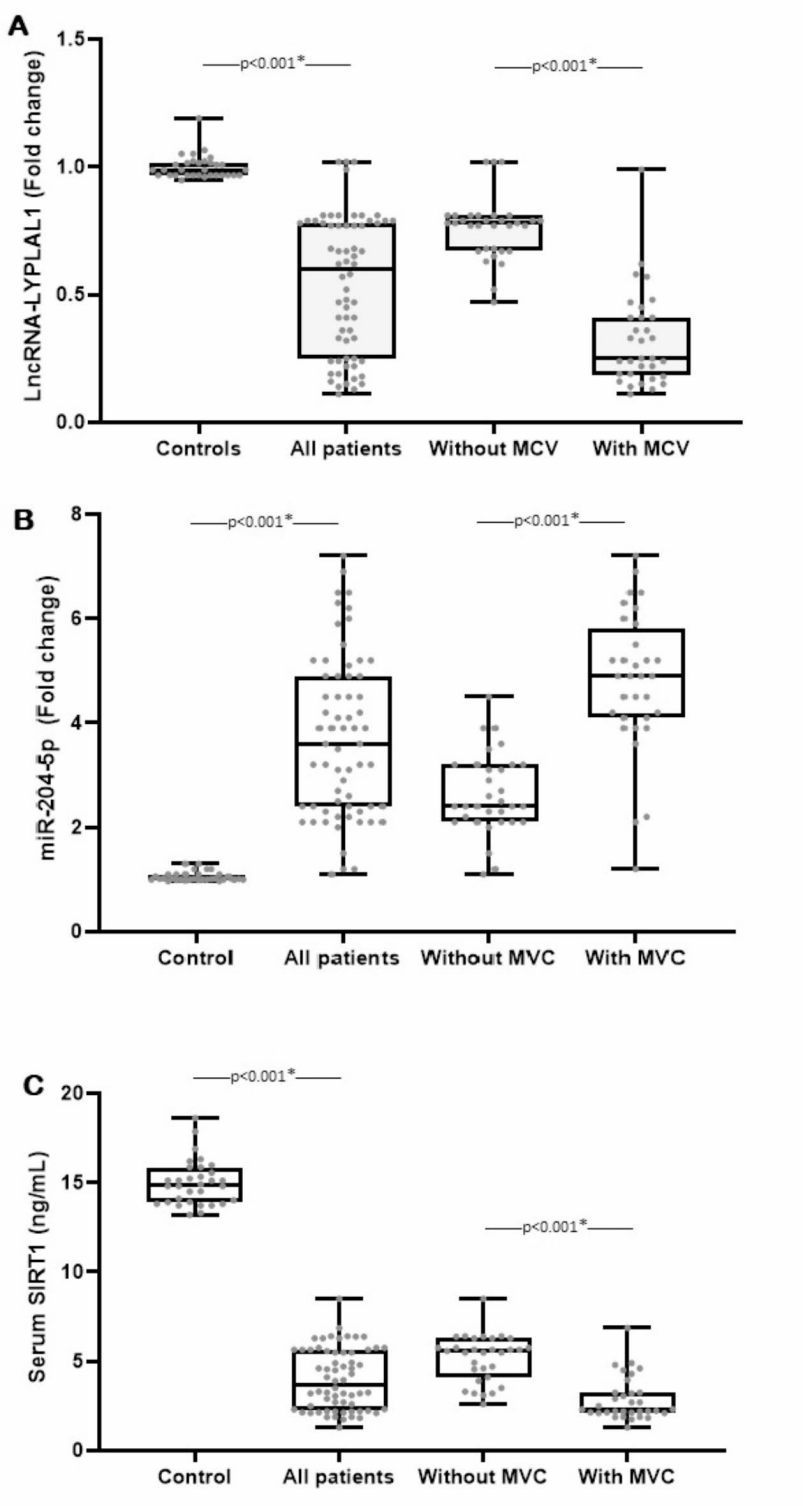
Between-groups comparison of (A) LncRNA LYPLAL1 expression, (B) miR-204-5p expression, and (C) SIRT1 levels. *MCV: Macrovascular complication*.

Raw Ct values for endogenous control, lncRNA LYPLAL1, and miR-204-5p were expressed in Table 2. When comparing cases with MVC to those without MVC, the lncRNA LYPLAL1 and SIRT1 values were significantly lower (P-value < 0.001). Conversely, patients with MVC had significantly higher miR-204-5p levels than those without MVC (P-value < 0.001) (Fig. 1).

**Table 1:** The characteristics of subjects.

Parameters	Controls (No.: 32)	Diabetic Patients (No.: 64)		*P*-value
		Patients without MVC (No.: 32)	Patients with MVC (No.: 32)	
**Age** (years)	44.5 [33.5–59]	48.5 [37.5–67]	48 [37–66]	0.98
**Gender**: Male/Female	22/10 (68.8/31.2)	27/15 (84.4/15.6)	23/9 (71.9/28.1)	0.31
**BMI** (kg/m^2^)	26.3 [23.6–31.4]	26.6 [23.3–29.5]	25.9 [23-29.5]	0.37
**Duration of diabetes** (years)	------	5.5 [2–16]	7.2 [2–17]	0.12
**Treatment**				
• Oral hypoglycemic drugs	------	26 (81.2)	22 (68.8)	0.25
• Insulin	------	6 (18.8)	10 (31.2)	
**Macrovascular complications**				
• CAD	------	------	20 (62.5)	
• PVD	------	------	4 (12.5)	
• CVD	------	------	8 (25)	
**Laboratory tests**				
• Fasting glucose (mg/dL)	81 [70–93]	192.3 [163–363] ^a^	203 [160–407] ^a^	< 0.001*
• Insulin (mU/L)	12.9 [6–16]	16.6 [9–25] ^a^	17 [9–25] ^a^	< 0.001*
• HOMA-IR	2.6 [1.2–3.4]	8.7 [4.5–16.6] ^a^	9.7 [4.51–23.1] ^a^	< 0.001*
• HbA1c (%)	5.2 [4.3–5.8]	8.6 [6.7–11.7] ^a^	9.7 [6.7–12.7] ^a, b^	< 0.001*
• Total Cholesterol (mg/dL)	123.6 [84–181]	204 [110–260] ^a^	199 [110–290] ^a^	< 0.001*
• Triglyceride (mg/dL)	79.7 [55.7-145.6]	139.3 [109.2–247] ^a^	143 [109–336] ^a^	< 0.001*
• HDL-C (mg/dL)	44.8 [31.4–67.3]	36.8 [26.5–55.6] ^a^	33.6 [24.5–44.3] ^a, b^	< 0.001*
• LDL -C(mg/dL)	63.6 [24.7–121]	148 [50.7-191.6] ^a^	144 [55-205.7] ^a^	< 0.001*

MCV: Macrovascular complication; BMI: Body mass index; CAD: Coronary artery disease; CVD: Cerebrovascular disease; PVD: Peripheral vascular disease (PVD); HOMA-IR: Homeostatic model assessment for insulin resistance; HbA1c: Hemoglobin A1c HDL-C: High-density lipoprotein cholesterol; LDL-C: Low-density lipoprotein cholesterol.

Data are expressed as median [range] or number (%).

p: Significance of Chi-square or Mann Whitney or Kruskal–Wallis H test then corrected Dunn’s test.

a: The significant difference in comparison to controls group.

b: The significant difference in comparison to patients without MVC group.

*: Significant.

**Table 2:** Raw ct values for endogenous control, LncRNA LYPLAL1, and miR-204-5p.

Parameters	Controls (No.: 32)	Diabetic Patients (No.: 64)	
		Patients without MVC (No.: 32)	Patients with MVC (No.: 32)
Endogenous control CT	21.09 [20.14–22.03]	20.9 [20.4–21.5]	21.4 [20.9-21.98]
LncRNA LYPLAL1 CT	25.18 [24.16–26.19]	25.37 [24.52-26]	27.36 [25.2–28.7]
miR-204-5p CT	27.92 [26.89–28.83]	26.36 [25.42–27.85]	25.93 [25.04–27.81]

Data are expressed as median [range]; CT: Cycle threshold.

### Diagnostic performance of the markers

**Fig. 2:**
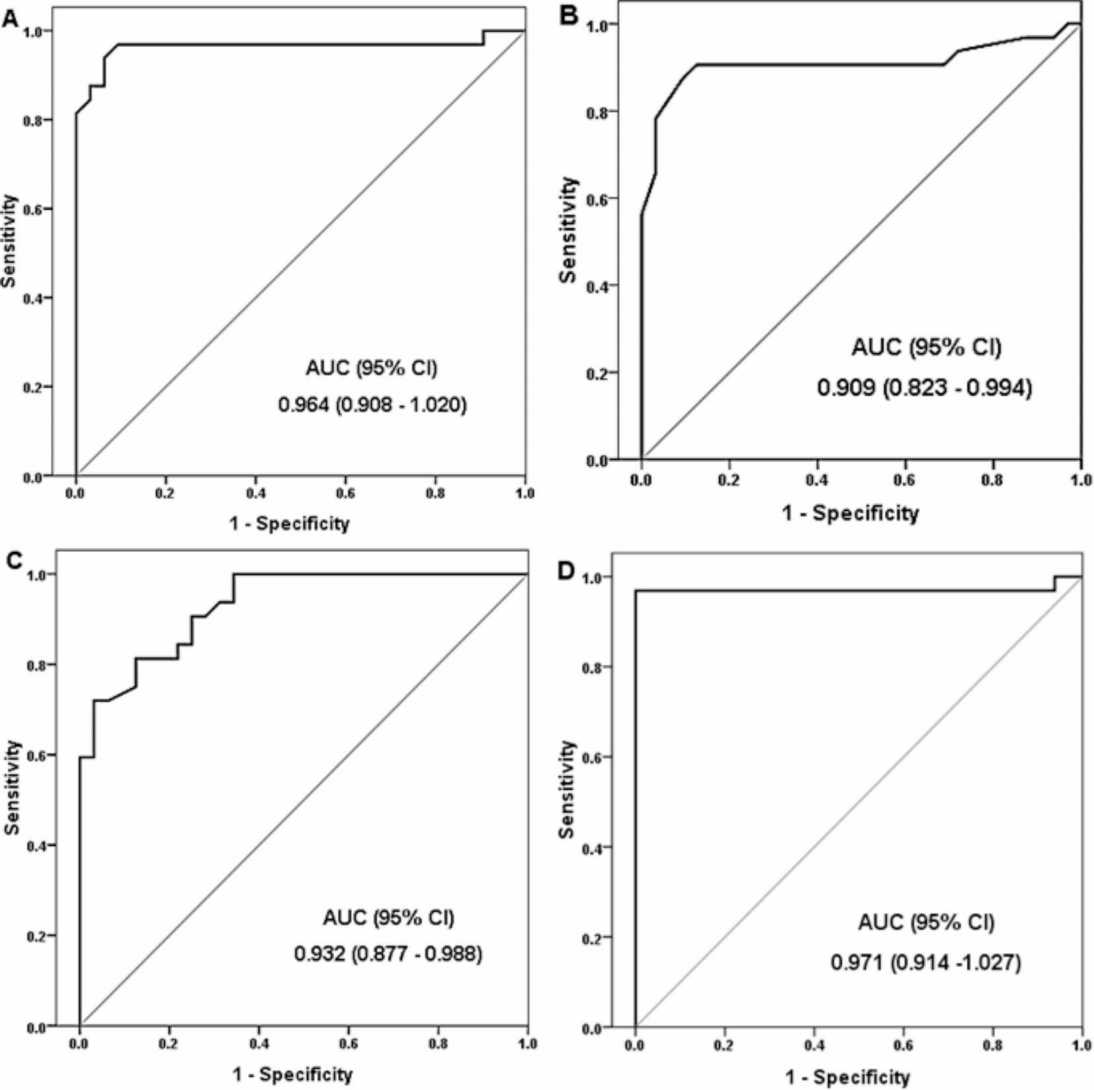
ROC curve analysis of the prediction of MVC in diabetic patients (A) LncRNA LYPLAL1 expression, (B) miR-204-5p expression, and (C) SIRT1 levels. (D) combined lncRNA LYPLAL1, miR-204-5p, and SIRT1.

The ROC curves for the prediction of MVC in patients with diabetes are shown in Fig. 2. The markers’ diagnostic capabilities were evaluated. LncRNA LYPLAL1, miR-204-5p, and SIRT1 had Youden indices of 0.88, 0.78, and 0.68, in that order (Table 3). The LncRNA LYPLAL1 performed best in terms of detecting MVC. It attained 90.6% specificity and 96.9% sensitivity. It also had an accuracy of 93.8%, a negative predictive value (NPV) of 96.7%, and a positive predictive value (PPV) of 91.2%. The best accuracy was obtained with a combination of three markers (lncRNA LYPLAL1, miR-204-5p, and SIRT1) at 98.4%.

**Table 3:** Markers’ diagnostic performance criteria.

Marker	Cutoff	Youden’s index	Sensitivity (%)	Specificity (%)	PPV (%)	NPV (%)	Accuracy (%)
LncRNA LYPLAL1	≤ 0.62 (fold change)	0.87	96.9	90.6	91.2	96.7	93.8
miR-204-5p	≥ 3.75 (fold change)	0.78	87.5	90.6	90.3	87.9	89.1
SIRT1	≤ 3.27 (ng/mL)	0.68	81.3	87.5	86.7	82.4	84.4
Combined lncRNA LYPLAL1, miR-204-5p, and SIRT1.	At same cutoffs	0.97	96.9	100	100	97	98.4

### Correlation analysis of the studied markers

Table 4 presents the correlation study. Except for HDL-C, which demonstrated a positive link, there were negative relationships found between the lncRNA LYPLAL1 and SIRT1 with glycemic profile and lipid profile. miR-204-5p exhibited negative connections with HDL-C but positive associations with other lipids and glycemic profiles. While there was a positive correlation between the two, the lncRNA LYPLAL1 and SIRT1 exhibited a negative correlation with miR-204-5p.

**Table 4:** Correlation analysis of the studied markers.

Parameters	LncRNA LYPLAL1		miR-204-5p		SIRT1	
	*r*_s_	*P*-value	*r*_s_	*P*-value	*r*_s_	*P*-value
Age	0.04	0.73	0.01	0.98	-0.09	0.41
BMI	0.01	0.94	0.03	0.81	0.01	0.98
Duration of diabetes	0.17	0.18	0.09	0.5	0.02	0.85
Fasting glucose	− 0.48	< 0.001*	0.48	< 0.001*	− 0.41	< 0.001*
Insulin	− 0.25	0.016*	0.26	0.01*	− 0.22	0.029*
HOMA-IR	− 0.45	< 0.001*	0.44	< 0.001*	− 0.39	< 0.001*
HbA1c	− 0.55	< 0.001*	0.54	< 0.001*	− 0.43	< 0.001*
Total Cholesterol	− 0.35	0.001*	0.37	< 0.001*	− 0.32	0.001*
Triglyceride	− 0.31	0.002*	0.38	< 0.001*	− 0.26	0.009*
HDL-C	0.34	0.001*	− 0.31	0.02*	0.27	0.006*
LDL -C	− 0.36	< 0.001*	0.36	< 0.001*	− 0.33	0.001*
miR-204-5p	− 0.84	< 0.001*	1	-------	− 0.74	< 0.001*
SIRT1	0.71	< 0.001*	− 0.74	< 0.001*	1	-------

rs: Spearman’s rank correlation coefficient; BMI: Body mass index; HOMA-IR: Homeostatic model assessment for insulin resistance; HbA1c: Hemoglobin A1c HDL-C: High-density lipoprotein cholesterol; LDL-C: Low-density lipoprotein cholesterol; SIRT1: Sirtuin 1.

### Logistic regression analysis of MVC risk factors

Utilizing binary logistic regression analysis, the risk factors for MVC were evaluated (Table 5). HbA1c, lncRNA LYPLAL1, miR-204-5p, and SIRT1 were linked to MVC prediction in the univariate study. No associations were seen with other factors. The multivariate model incorporating all variables listed in Table 4 nevertheless shows a significant correlation between MVC and lncRNA LYPLAL1 expression. They appear to be an independent MVC predictor. Adjusted OR for LYPLAL1 expression was 405 (95% CI: 1.4–1200) (P-value = 0.039).

**Table 5:** Logistic regression analysis of MVC risk factors.

Variables	Univariate		Multivariate	
	OR (95% CI)	*P*-value	AOR (95% CI)	*P*-value
Age	1 (0.4–1.07)	0.93	0.96 (0.76–1.2)	0.73
BMI	0.92 (0.67–1.28)	0.63	0.39 (0.07–2.3)	0.30
Duration of diabetes	1.12 (0.97–1.29)	0.12	1.59 (0.84-3)	0.15
HbA1c	1.49 (1.0-2.09)	0.02*	1.35 (0.48–3.8)	0.57
LDL-C	1 (0.99–1.02)	0.52	1.01 (0.97–1.05)	0.83
LncRNA LYPLAL1	225 (29.7–1700)	< 0.001*	405 (1.4–1200)	0.039*
miR-204-5p	67.7 (13.8-329.9)	< 0.001*	7.4 (0.2–260)	0.27
SIRT1	79.2 (9.4-670.1)	< 0.001*	513 (0.3–1500)	0.10

OR: Odds ratio; AOR: Adjusted odds ratio; CI: Confidence interval; BMI: Body mass index; HbA1c: Hemoglobin A1c; LDL-C: Low-density lipoprotein cholesterol; SIRT1: Sirtuin 1.

## Discussion

Diabetes is a significant global health issue. Both macrovascular and microvascular disorders have their primary cause^27^. A study was conducted to screen lncRNA profiles of leukocytes from diabetic MVC patients in order to investigate the protective impact of lncRNA LYPLAL1-DT on EC derived from human umbilical vein endothelial cells cultured under high glucose, hyperoxia, and inflammatory conditions. When primary human umbilical vein endothelial cells (HUVECs) were treated with exosomes obtained from diabetic MVC patients’ serum, miR-204-5p expression increased compared to the control. Exosomes produced from diabetic MVC patients’ serum significantly reduced HUVEC viability and migration compared to control. SIRT1 expression was elevated in EC but lowered by the miR-204-5p mimic. A dual luciferase reporter test revealed that SIRT1 is miR-204-5p’s binding target gene^19^.SIRT1 is a crucial player in the control of oxidative stress, glucose/lipid metabolism, insulin resistance, inflammation, and mitochondrial activity. Additionally, SIRT1 controls the amount of insulin secreted by β-cells and shields them from tissue damage caused by oxidative stress and inflammation. Major SIRT1 activators have been shown to have a positive effect on reversing problems associated with diabetes^28^. These findings are consistent with previous observations that SIRT1 decreases monocyte adherence to the vascular endothelium and participates in the inhibitory effects of endothelial cell death. More critically, we verified the most recent finding that miR-204-5p/SIRT1 promotes inflammation and endothelial cell dysfunction^29^.

In this study, SIRT1 levels were considerably diminished in patients with MVC in comparison to those without MVC. The finding that hyperglycemia reduces SIRT1 expression and speeds up endothelium senescence provided an explanation for similar outcomes. In diabetic mice, hyperglycemia-induced endothelium senescence is inhibited by SIRT1 activation, protecting against vascular impairment. Vascular problems result from hyperglycemia’s reduction of Sirt1 expression^30^. Furthermore, a different study found high hyperglycemia results in SIRT1 downregulation and lowered SIRT1 expression^31^. In muscle biopsies obtained from T2DM individuals, Kitada et al.^21^ showed lower expression of the SIRT1 protein. Since type 2 diabetic patients and controls did not differ in SIRT1 mRNA levels, this impact was most likely caused by post-transcriptional changes.

MicroRNA regulates its target messenger RNA (mRNA) post-transcriptionally through mRNA degradation or translational repression, which in turn affects the target mRNAs’ expression through post-transcriptional suppression of the translation process^32^. By controlling the expression of several genes, miRNAs have been shown to have a potential role, either direct or indirect, in a number of disorders. According to Mao et al.^33^, there was evidence of upregulation of miR 204 5p in the retina tissues of diabetic rats, which could potentially be linked to the development of diabetic retinopathy.

In this study, when comparing cases with MVC to those without MVC, the lncRNA LYPLAL1 and SIRT1 values were significantly lower. Our study demonstrated that patients with MVC had significantly higher miR-204-5p levels than those without MVC. On the other hand, a study found that patients with low circulating miR-204 levels were at a higher risk of CVD. After accounting for relevant confounders, miR-204 was independently related to CVD in patients with type 2 diabetes^34^. Also, Cao et al.^35^ found overexpression of miR-204-5p reduced reactive oxygen species levels, pro-apoptosis genes, and inflammatory cytokines production in high glucose-treated human lens epithelial cells. This is not agreed with Gok et al.^36^, who reported that SIRT1 protein levels significantly increased in diabetic patients compared to those in the control group. However, SIRT1 expression in peripheral blood mononuclear cells correlates strongly with inflammatory cytokine levels in individuals with coronary artery disease and type 2 diabetes, but not with the severity of coronary lesions^37^. The sample size and demographics of the study populations may vary significantly, leading to differences in results. The severity and progression of diabetes and its related macrovascular complications may vary significantly between study populations, leading to differences in biomarker levels.

A prior study found that miR-204-5p plays a direct role in the regulation of SIRT1 in diabetic corneas and that this regulation of SIRT1 is mediated by miR-204-5p, which in turn delays the traversal from the perspective of the epithelial cell cycle in diabetic keratopathy. Among these miRNAs, it was shown that diabetic corneas had an almost 5.0-fold increase in miR-204-5p expression compared to the non-diabetic control. Transfection with a miR-204-5p inhibitor enhanced SIRT1 protein expression, while transfection with a miR-204-5p mimic decreased it. Nevertheless, neither the overexpression nor the inhibition of miR-204-5p significantly affected the levels of SIRT1 mRNA, indicating that miR-204-5p predominantly reduced SIRT1 expression at the post-transcriptional stage. Corneal epithelial cells expressed more miR-204 when they were hyperglycemic. Furthermore, they demonstrated that an increase in SIRT1 in conjunction with miR-204 suppression could counteract the adverse consequences of hyperglycemia^38^. A prior study provided an explanation for similar findings, showing that the retinal tissue of diabetic rats had considerably higher levels of miR 204 5p as compared to the control group. This finding suggested that miR 204 5p could be linked to diabetes and accelerate the development of retinopathy^33^. Similarly, Zhang et al.^39^ discovered that miR-204 downregulates SIRT1 in gastric cancer cells.

Also, when we compared cases with MVC to those without MVC, the lncRNA LYPLAL1 values were significantly lower. Diabetes with persistently elevated blood glucose will eventually cause inflammation and blood vessel stimulation, which will result in MVC. LncRNAs have become important modulators in various processes that are either physiological or pathological. All the findings supported the importance of circulating leukocyte lncRNAs to the pathophysiology of MVC^40^. The chosen lncRNA, LYPLAL1-DT, demonstrated protective properties for endothelial cells. In terms of mechanism, LYPLAL1-DT functions as a competitive endogenous RNA (ceRNA) by suppressing miR-204-5p, which in turn increases SIRT1 and strengthens the anti-inflammatory response^14^. Leukocytes are the source of LYPLAL1-DT, which is expressed at a lower level in MVC patients’ serum than in healthy individuals. LYPLAL1-DT expression was four times lower in MVC cells than in healthy samples, according to exosome sequencing. Moreover, LYPLAL1-DT is a significant prospective target for MVC studies.

Further proof of their protective benefits was discovered when it was shown that overexpression of LYPLAL1-DT might increase the release of anti-inflammatory cytokines^17^. These results demonstrated that circulating leukocyte-derived lncRNAs play a critical role in the pathophysiology of MVC and that LYPLAL1-DT, through the LYPLAL1-DT-miR-204-5p/SIRT1 pathway, prevents against damage to vascular endothelium in complications related to diabetes^19^. According to Chang et al.^18^, LYPLAL1-DT protects endothelial cells from damage in inflammatory and hyperglycemic circumstances.

It was evaluated to see how well these markers predicted MVC. LYPLAL1 LncRNA demonstrated the best performance. It achieved 96.9% sensitivity and 90.6% specificity. Its accuracy rate was 93.8% as well. Combining the three markers (lncRNA LYPLAL1, miR-204-5p, and SIRT1) had the greatest accuracy, which was 98.4%. It appears that lncRNA LYPLAL1 expression is an independent predictor of MVC. Comparing circulating lncRNAs to traditional glycoprotein markers, they have demonstrated improved diagnostic effectiveness^41^.

The lncRNAs LYPLAL1 and SIRT1 were discovered to have negative correlations with glycemic profile and lipid profile, except for HDL-C, which showed a positive link. miR-204-5p showed positive correlations with the glycemic profile and other lipids but negative correlations with HDL-C. There was a positive link between the lncRNAs SIRT1 and LYPLAL1 and a negative correlation between them and miR-204-5p. Adipose-derived mesenchymal stem cells’ adipogenic differentiation is positively regulated by the lncRNA LYPLAL1-AS1. According to Yang et al.^42^, LYPLAL1-AS1 could be a novel therapeutic target for minimizing and management of disorders that are caused by aberrant adipogenesis. In their 2020 study, Wang et al.^43^ examined the function and mechanism of microRNA-204-5p in atherosclerosis and discovered that, in contrast to normal controls, The level of miR-204-5p expression in atherosclerotic plaque and peripheral blood was significantly reduced. SIRT overexpression in diabetes may lead to better control of glucose and lipid profiles.

There are several reasons why a correlation between fasting blood glucose and MCV prediction was not found, such as MCV develops over a long period of time, whereas fasting blood glucose is typically measured at a single point in time, and the compensatory hyperinsulinemia can lead to normal or even low fasting blood glucose levels, despite the presence of insulin resistance and increased risk of macrovascular complications^44^. There are several reasons why a correlation between lipid profile and MCV prediction was not found, such as lipid profiles are not the sole determinant of MCV, it influenced by a variety of factors, including blood pressure, smoking, physical activity, diet, and family history. Also, inflammation and oxidative stress can contribute to the development of MCV, regardless of lipid profiles. However, lipid profiles can be affected by factors such as medication use (e.g., statins), hormone replacement therapy, and certain medical conditions (e.g., kidney disease)^45,46^.

The study’s design revealed some limitations. Notably, this research was conducted at a single center. Secondly, there was no assessment of how the medication affected the markers’ levels. Lastly, a need for additional assessments to generalize the findings.

## Conclusions

When we compared cases with MVC to those without MVC, the lncRNA LYPLAL1 and SIRT1 values were significantly lower. Patients with MVC had significantly higher miR-204-5p levels than those without MVC. LYPLAL1 LncRNA demonstrated the best performance characteristics. lncRNA LYPLAL1 expression is an independent predictor of MVC. Consequently, due to its benefits, we may infer that lncRNA LYPLAL1 expression is an independent predictor of MVC that could represent a safe, viable future marker for diabetes and associated macrovascular complications.

## Data Availability

Data will be available on request to Dr. Marwa M. Esawy, Clinical Pathology Department, Faculty of Human Medicine, Zagazig University, Egypt.
